# Book Notices

**Published:** 1857-03

**Authors:** 


### BOOK NOTICES.

*Transactions of the American Medical Association, Vol. IX.* 1856. Philadelphia: Printed for the Association by T. R. & P. G. Collins.

(Continued from Feb. No.)

The next paper in the volume before us, and closely allied to that of Dr. Gross, which was noticed in the previous number of the Journal, is the Report from the regular Standing Committee on Medical Literature.

It occupies thirty pages, and was written by Dr. R. J. Breckenridge of Kentucky, Chairman of the Committee; and is signed by two of his colleagues, Drs. J. B. Flint and D. L. McGugin. This paper presents some excellent qualities, but so mingled with defects as to greatly lessen its value. As a defence, we might almost say *eulogy*, of American Medical Literature; as an argument against International Copywright laws, and in favor of the common brotherhood of the profession and the universality of its literature, without regard to geographical lines or nationalities, and as a specimen of the author's ease and elegance of style as a writer, it is worthy of the highest commendation.

As a report, however, on the Medical Literature of this country, under a constitutional rule requiring the Committee to examine the medical periodicals and embrace in the report a 'reference to the more important articles therein presented to the profession,' together with such 'original American medical publications and American reprints of foreign works,' as have been issued during the year of the Committee's service, it is extremely defective and unsatisfactory; being neither creditable to the industry or good judgment of the author. For instance, the very important duty of examining the medical periodicals of the country is entirely omitted, under the plea of 'not having had access to anything like complete files of the various journals of the country.' We do not believe there is an editor of a medical journal in the United States, who would not have cheerfully furnished the Chairman of the Committee a complete file of his journal for the year, had he taken the trouble to ask for it. There is no measure better calculated to improve medical journalism and encourage the better class of contributors, than the reviews contained in all the previous reports on medical literature, with a special enumeration of the more important articles. Again, the transactions of our National Association find their way to the libraries of public institutions and societies in Europe as well as this country, and everywhere the report on medical literature will be looked to for an exposition of the literary labors of the profession during the period it embraces. This consideration alone should have prevented the Committee from entirely neglecting so important a department of literature as the medical periodical press.

That part of the report devoted to the notice of original American publications is but little more satisfactory. In it we find half a page devoted to the crazy, advertising pamphlet of M. L. Knapp, on the Scorbutic Origin of Cholera, while the transactions of seven state medical societies are mentioned only by their *titles*, without an allusion to a single paper contained in them. Neither does the report fairly represent the character of those which had preceded it from other committees on the same subject. Thus Dr. Breckenridge says: 'It will be found that *scarcely a solitary reporter* to your body on this subject (medical literature) has failed to *lament* the low state and condition of our professional literature, and to suggest explanations more or less satisfactory of such condition, and means more or less profound of remedying it.' Again he says: 'From the *dignified reporters* of the Association to the silliest of anonymous scribblers, who *bewail with grievous lamentations* this national degradation;' &c. These paragraphs, together with the whole tenor of the report before us, would lead the reader to infer that all the previous committees on medical literature had united in depreciating and *bewailing* the condition of the medical literature of our country; than which nothing could be more untrue. Will Dr. B. point us to a single word of 'lamentation' in the report on this subject, made by the late Dr. Harrison, of Cincinnati, to the annual meeting of the Association in Boston; or in that made by Dr. Reyburn, of St. Louis, to the meeting in Charleston; or that made by myself to the second meeting in New York? A reference to those reports will show that their authors, instead of indulging in depreciating remarks or lamentations, exhibited as high and liberal an appreciation of the merits of our professional literature as Dr. Breckenridge himself. It is true that this appreciation did not prevent them from pointing out its obvious defects with manliness and candor. And, had it been any part of their duty, they would have pointed out defects scarcely less important in the literature of every other nation in Christendom.

If Dr. Oliver Wendell Holmes, of Boston, and Dr. Alfred Stille, of Philadelphia, in their respective reports to the Association, indulged in language *too depreciative* of our medical literature, let them be responsible, without including all the reporters on this subject in the same category.

But even the author of the report under consideration admits that our literature is not yet faultless or complete; for at the close he asks the following questions, viz:—

'What then can be done to correct the faults of our medical literature, however great or however small they may be? Is there a remedy?' And to these he furnishes the following laconic answers. 'In the course of time and through the unchangeable laws of progress and development—yes. Through the agency of this Association? No.' In one sense these answers may be considered correct. A refined philosophy might easily claim that the correction of all faults, the advancements of every kind, whether in morals, science, or religion, are but the legitimate consequence of 'the unchangeable laws of progress and development.' In this view the American Medical Association is itself a product of these laws, a development of the times, a result of the spirit of combination and associated action so characteristic of the middle of the nineteenth century. But our reporter and all others should remember, that even the '*unchangeable laws of progress*' are applied and rendered effective *only* through *agencies* or instrumentalities. And of all the agencies capable of appreciating and applying the laws of progress to the development of our medical literature, none can be found more efficient than our National Association. Not by formal resolves, but by its annual re-union; its faithful reviews contained in the reports of its committees; its prizes; its strong incentives to action, carried by its members into all the local organizations throughout our widely-extended national domain.

The next paper in the transactions claiming our attention, is the 'Report of the Committee on Plans of Organization for State and County Societies,' written by Dr. A. B. Palmer, of this city. It is a well-written plea in favor of a more complete organization of the profession, by the formation of State and County Medical Societies. Its doctrines are very fully presented in the following resolutions, which we copy entire:—

'1. *Resolved,* That the American Medical Association, appreciating the vast benefits to the advancement of medical science, to the profession, and to the interests of humanity, arising from the efficient organization of medical men, call with deep earnestness upon physicians everywhere throughout the country, to form themselves into County and State Medical Societies.

'2. *Resolved,* That this Association earnestly recommend to all County Medical Societies to incorporate into their constitutions or by-laws provisions for the election of a Board of Censors, whose duty it shall be to examine all persons who may apply for admission into the office of any member of the society as students of medicine, and also to incorporate provisions to prevent any member of such society from admitting as a student any person who shall not first receive from the Board of Censors a certificate of a good moral and intellectual character; a good English education, including a thorough knowledge of the English language, and a respectable acquaintance with its literature, and with the art of composition; a fair knowledge of the natural sciences, and at least the more elementary mathematics, including the chief elements of algebra and geometry, and such a knowledge of the ancient languages as will enable him to read current prescriptions, and appreciate the technical language of the natural sciences and of medicine.

'3. *Resolved*, That this Association also earnestly recommend to local or county societies to incorporate into their constitutions or by-laws provisions for making it the duty of each of the members to keep at least a brief record of all cases occurring in his practice depending upon endemic or general causes, and report at least annually to a committee of the society to which he belongs the number or per centage of different diseases occurring each month, together with the particular type of each disease, the chief modifying circumstances under which it occurred, the general plan of treatment, and the results of the cases; and also, that these societies make provision for the election of such committee, whose duty it shall be to receive and collate such reports, arranging them in due form, and adding such remarks as may assist to their proper understanding, and to transmit them annually thus arranged to a committee of the State society to which the local or county society shall be auxiliary; and this Association further recommends that the State societies make provision in their constitutions or by-laws for the appointment of a committee, whose duty it shall be to receive such reports from the local or county societies, to again arrange them with other reports from similar societies, placing them in a condensed or tabulated form, and report them annually with proper remarks to a committee of this Association, to which the State societies are recommended to become auxiliary.

'4. *Resolved*, That this Association make provision for the reception of these reports from the State societies, by a committee or committees, whose duty it shall be to arrange them in proper form, adding such illustrative remarks as may be deemed proper, and to report them to this Association, with a view of having them published with the other transactions of this body.

'5. *Resolved*, That this Association recommend the adoption of its Code of Medical Ethics to all societies auxiliary to it, and that they record or publish said code with their constitutions and by-laws.

'6. *Resolved*, That, in the opinion of this Association, it would tend to the production of papers of greater merit, and increase the interest of the meetings of local or county societies, if those papers possessing peculiar merit were referred to the State Society as marks of honor, and to be incorporated into their proceedings, if deemed worthy.

'7. *Resolved,* That, in arranging the details of the practical workings of societies, due attention should be paid to a proper division of labor, special subjects for investigation, and reports being referred to members who, from their mental aptitudes, their previous studies, or their peculiar opportunities or positions, are best qualified and most inclined to do them justice.'

It strikes us that the plan of reporting embraced in resolutions 3 and 4, is too complicated, and involves too much delay before final publication, to be readily reduced to practice. We have no objections to seeing it tried however.

The next paper in order, is the 'Report on the Changes in the Composition and Properties of the Milk of the Human Female, produced by Menstruation and Pregnancy,' by the editor of this *Journal.* It was republished at length in the December number of this *Journal*, and consequently needs no further notice here.

(To be Continued in our next No.)

---

*The Half-Yearly Abstract of the Medical Sciences, &c.* Edited by W. H. RANKING, M.D. Physician to the Norfolk and Norwich Hospital; and C. B. RADCLIFFE, M.D. Licentiate of the Royal College of Physicians of London. No. 24, from July to December, 1856. Philadelphia, Lindsay & Blakiston, 1857.

This, like Braithwait's Retrospect, is too well known to the medical public to require notice in this place. The present number contains 276 pages of very useful reading matter.

---

*Fourth Annual Report of the Surgeons of the New York Opthalmic Hospital, at No. 6. Stuyvesant Place, with the Anniversary Address of* ISAAC FERRIS, D.D., LL.D. &c. &c. Daniel Fanshaw, Printer, New York, 1857.

We have noticed the New York Opthalmic Hospital in former numbers of this *Journal.* The present report bears evidence of the continued prosperity and usefulness of the institution.

The Address of Dr. Ferris is short, but interesting and valuable.

*An Address on the Life and Character of* ROBERT M. PORTER, M.D. *Late Professor of Anatomy in the University of Nashville.* By JOHN BERRIEN LINDSLEY, M.D. Chancellor of the University. Delivered at Nashville, Nov. 8, 1856. Published by the Class.

This is a beautifully-written eulogy of the late Dr. Porter, and as beautifully printed as it is written.

